# FPGA-Based Feature Extraction and Tracking Accelerator for Real-Time Visual SLAM

**DOI:** 10.3390/s23198035

**Published:** 2023-09-22

**Authors:** Jie Zhang, Shuai Xiong, Cheng Liu, Yongchao Geng, Wei Xiong, Song Cheng, Fang Hu

**Affiliations:** 1 National Astronomical Observatories, Chinese Academy of Sciences, Beijing 100101, China; 2 The 20th Research Institute of China Electronics Technology Group Corporation, Xi’an 710068, China; 3 CETC Galaxy BEIDOU Technology (Xi’an) Co., Ltd., Xi’an 710061, China; 4 Beijing Eyestar Technology Co., Ltd., Beijing 102200, China

**Keywords:** VIO, V-SLAM, FPGA, histogram equalization, FAST, pyramid processing

## Abstract

Due to its advantages of low latency, low power consumption, and high flexibility, FPGA-based acceleration technology has been more and more widely studied and applied in the field of computer vision in recent years. An FPGA-based feature extraction and tracking accelerator for real-time visual odometry (VO) and visual simultaneous localization and mapping (V-SLAM) is proposed, which can realize the complete acceleration processing capability of the image front-end. For the first time, we implement a hardware solution that combines features from accelerated segment test (FAST) feature points with Gunnar Farneback (GF) dense optical flow to achieve better feature tracking performance and provide more flexible technical route selection. In order to solve the scale invariance and rotation invariance lacking problems of FAST features, an efficient pyramid module with a five-layer thumbnail structure was designed and implemented. The accelerator was implemented on a modern Xilinx Zynq FPGA. The evaluation results showed that the accelerator could achieve stable tracking of features of violently shaking images and were consistent with the results from MATLAB code running on PCs. Compared to PC CPUs, which require seconds of processing time, the processing latency was greatly reduced to the order of milliseconds, making GF dense optical flow an efficient and practical technical solution on the edge side.

## 1. Introduction

With visual odometry (VO) and visual simultaneous localization and mapping (V-SLAM) technologies, the carrier can achieve location, navigation, and mapping in environments with GNSS signal occlusion and rejection. This makes VO and V-SLAM the core and key technologies in applications such as autonomous driving, robotics, unmanned aerial vehicles (UAV), and virtual reality (VR) [1,2,3,4,5]. Compared with other autonomous positioning methods such as LiDAR (Light Detection and Ranging) and inertial measurement unit (IMU), the main challenge of visual-based solutions comes from computing power. Due to the need for fast, stable, and reliable processing of high frame rate and high-resolution image stream data, VO and V-SLAM systems typically require high-performance computing platforms, which greatly increases equipment costs and limits the further large-scale application of the technology.

The above problems can be effectively solved by using chips or processors specially designed for complex tasks such as image processing to achieve the purpose of hardware acceleration [6,7,8,9]. At present, GPU and FPGA are two representative hardware acceleration technology routes, and both are widely used in the field of image processing. In contrast to CPUs and GPUs based on the von Neumann architecture, the function of each logic unit of an FPGA is determined when reprogrammed without instructions or shared memory for communication [10]. Therefore, for streaming computing tasks, FPGA has inherent advantages in latency and higher energy efficiency. In addition, FPGA is very flexible in use, which can change hardware algorithms and chip functions to facilitate algorithm and function verification. Therefore, it can serve as both a product and a prototype platform for chip design. Due to the above advantages, FPGA has been increasingly widely used in the field of VO and V-SLAM technology in recent years.

R. Taranco et al. (2021) designed a FPGA-based Oriented FAST and Rotated BRIEF (ORB) feature extraction for self-driving [11]. In the process of calculating the rBRIEF descriptor, a scheduling technique based on a genetic algorithm was used. Compared with CPU systems, this accelerator achieves image processing acceleration while greatly reducing power consumption, but does not support optical flow computing. A real-time multi-scale Lucas Kanade (LK) optical flow hardware accelerator with parallel pipeline architecture has been proposed [12]. It was deployed on a Xilinx Zynq SoC and achieves a frame rate of 93 FPS for feature tracking of continuous frame images at 752 × 480 resolution. T. Stúrmanis and R. Novickis (2021) developed a FPGA-based optical flow computing accelerator based on FAST feature detection and BRIEF feature descriptors [13]. By dividing the image into static regions, feature points are tracked between frames. The accelerator is fully pipelined and achieves a performance of 300 frames per second through VGA resolution images, but it also only supports sparse optical flow calculation and tracking. Navion is a real-time VIO accelerator for nano drones [14]. The entire VIO system is the first to be fully integrated on a chip, which is fabricated in 65 nm CMOS and can process 752 × 480 stereo images from the EuRoC dataset in real-time at 20 FPS [15]. However, because it is specifically designed for micro-drone applications, Navion compromises on many fronts. For example, it can only support 480P images, and reduces character length and limits the number of feature points to compress memory. In addition, many of its hardware parameters are determined based on the simulation analysis of the EuRoC dataset, which also limits the flexibility and universality of its application to a certain extent. Chiang-Heng Chien et al. (2021) proposed a multiple master-slave FPGA architecture for a scale-invariant feature transform (SIFT)-based stereo VO [16]. The master-slave design enables high reconfiguration for the data throughputs among various modules such as SIFT and matching. In the SIFT module, a hardware implemented image pyramid was proposed, where scales were determined off-line via a minimization approach. Local linear exhausted search (LES) matching was used for both the stereo and the frame matching. This achieved a frame rate of 33.2 frames per second. The first CMOS-based dedicated hardware accelerator was proposed (HcveAcc) byLi Renwei et al. (2020), which was implemented in 28 nm CMOS technology using commercial EDA tools [17]. HcveAcc solved the time-cost bottleneck in the tracking process-high-density feature extraction and high-precision descriptor generation. Compared with state-of-the-art FPGA solutions, HcveAcc achieves 4.3× speedup while consuming much less energy. Jingyuan Li et al. (2022) developed a FPGA-based high-throughput keypoint detection accelerator using convolutional neural networks (CNN) with algorithm–hardware co-design, including a lightweight keypoint detection neural network and a dedicated hardware accelerator architecture [18]. Implemented on a Xilinx ZCU104 FPGA board, the proposed accelerator is able to perform keypoint detection at 94 FPS for a 640 × 480 input image, with fast processing speed.

In this paper, a FPGA hardware acceleration solution for VO and V-SLAM application is proposed and implemented on a Xilinx Zynq FPGA (UltraScale+ MPSoC ZU15EG). The proposed accelerator consists of an image preprocessing module, pyramid processing module, optical flow processing module, and feature extraction and tracking module, and realizes the complete acceleration processing function of the image front-end and directly outputs the feature point ID and coordinates to the back-end. Compared with other FPGA-based VO or V-SLAM acceleration solutions, the proposed accelerator adopts the contrast limited adaptive histogram equalization (CLAHE) algorithm with excellent performance to better improve the image preprocessing quality. For the first time, we have implemented a solution that combines FAST features with GF dense optical flow. Compared with the commonly used sparse optical flow, dense optical flow calculates the displacement of all pixels in the image and performs registration, resulting in better optical flow tracking performance. The implementation of edge-based acceleration for dense optical flow also provides a more flexible technical route for the backend—it can use FAST features and their corresponding optical flow for pose estimation, or directly use dense optical flow for estimation (known as the dense direct method). It also facilitates the creation of dense/semi-dense maps. In addition, to solve the scale invariance and rotation invariance lacking problems of FAST features, we designed and implemented a pyramid module with a five-layer thumbnail structure, and optimized its pipeline and memory read and write operations.

A test benchmark system was built, which could compare the processing results of the same image stream data on the FPGA side and on the PC side. The test results showed that the accelerator could achieve stable tracking of features of violently shaking images, and were consistent with the processing results from the MATLAB code on the PC side, which proves the effectiveness and correctness of the proposed real-time V-SLAM accelerator. In terms of hardware consumption, the proposed hardware system consumes 36% of the LUTs, 52% of the BRAM and 19% of the DSP of the Zynq FPGA (UltraScale+ MPSoC ZU15EG). In terms of throughput, when the accelerator operates at a frequency of 100 MHz, it can process 108 frames per second for 720P resolution images and 48 frames per second for 1080P resolution images. The accelerator can operate at a maximum of 200 MHz, further doubling the processing power. In terms of processing latency, for 720P resolution images, the accelerator takes about 10 ms per frame to calculate (operating at 100 MHz). Compared to PC CPUs that require seconds of time for processing, the processing latency was greatly reduced, making GF dense optical flow an efficient and practical technical solution on the edge side.

The remainder of the paper is structured as follows: Section 2 introduces the acceleration scheme, including the overall design and the GF dense optical flow tracking and FAST feature extraction methods. Section 3 presents the hardware architecture, which mainly consists of image preprocessing, pyramid processing, optical flow processing, and feature extraction and tracking modules. Finally, in Section 4, the implementation of the proposed accelerator is described, along with the evaluation based on the test benchmark system.

## 2. Acceleration Scheme

An overview of the proposed FPGA-based accelerator is firstly presented in this section, including its basic functionality and composition. On this basis, a brief introduction and review were conducted on the GF dense optical flow tracking and FAST feature method.

### 2.1. Overall Design

**Figure 1:**
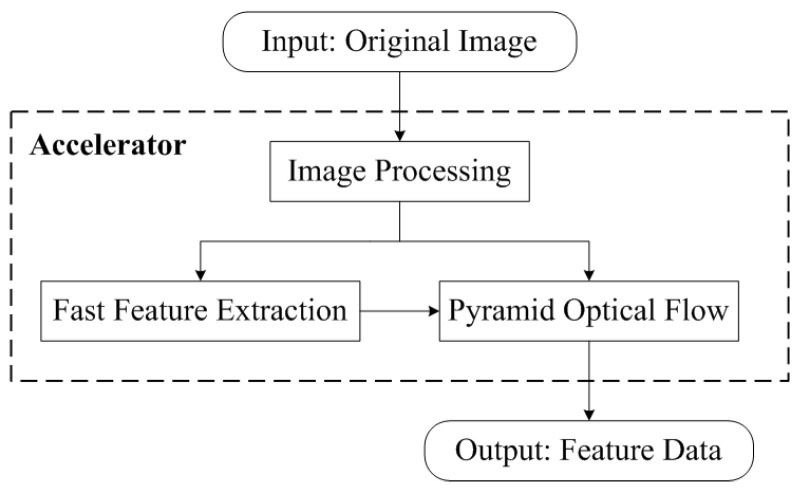
Overview of the acceleration function.

An overview of the FPGA-based accelerator is shown in Figure 1. The camera output data are transmitted to the FPGA through the MIPI interface, and the MIPI receiving module of the FPGA decodes the data into an AXI stream format for easy subsequent processing. The image is first subjected to histogram equalization processing, and then divided into two paths. One path is subjected to Gaussian blur processing, following which the pyramid is reduced and the optical flow calculation results are obtained layer by layer. The other path involves the extraction of FAST feature points and merging of the optical flow to update the results of the previous frame feature points. This allows the realization of the hardware acceleration functions of image preprocessing, FAST keypoint extraction, pyramid optical flow calculation and feature tracking, and directly outputs the ID and coordinate data of features to the back-end (that is, the FPGA PS side).

Unlike the commonly used method of combining feature points and sparse optical flow, this study adopts a dense optical flow scheme. Compared to the sparse optical flow, the dense optical flow calculates the displacement of all pixels in the image, forming a dense optical flow field [19,20,21]. However, its disadvantages are obvious. Since the displacement of all pixels needs to be calculated (typically several million pixels), the computation of the dense optical flow is significantly greater than that of the sparse optical flow, so it cannot be calculated in real-time on a CPU. Compared to a CPU, FPGA directly accesses the data stream captured by the camera via AXI (Advanced extensible Interface) Bus and calculates the optical flow in a streaming way, making it more efficient and faster.

The FPGA acceleration solution based on the mixing of dense optical flow and FAST features can bring us the following benefits:

- The dense optical flow field can be used for pixel-level image registration, so the optical flow tracking accuracy is significantly better than that of the sparse optical flow.
- The back-end can still use FAST features and their corresponding sparse optical flows for pose estimation or can directly use the dense optical flow for estimation, making it more flexible to use.
- The dense optical flow facilitates the construction of a complete map.

### 2.2. GF Dense Optical Flow Tracking

Optical flow refers to the displacement of target pixels in adjacent frame images caused by camera motion. Generally, sparse optical flow processing is faster and more suitable for low-end computing platforms [22]. Compared to sparse optical flow, dense optical flow does not only select image features for calculation but calculates the displacement of all pixels in the image, and performs registration based on the optical flow field, so the effect is better than sparse optical flow.

This work uses the GF dense optical flow to obtain better visual front-end performance, which is also a currently supported optical flow method in OpenCV [23,24]. With the support of FPGA architecture and resources, GF optical flow can run at a high frame rate in real-time, overcoming the limitation of computational power. In this way, it can be used independently for direct SLAM, as well as in conjunction with feature points, just like sparse optical flow, making it a solution with strong engineering practicality.

The principle of GF optical flow is to approximate some neighborhood of each pixel with a polynomial expansion. In this process, each pixel in the neighborhood no longer has the same influence on the central pixel, but instead uses a two-dimensional Gaussian distribution in a polynomial function to assign different weights to different pixels. Consider a quadratic polynomial, giving us the local signal model, expressed in a local coordinate system:

(1) $$\;f\left(x\right)\sim x^{T}Ax+b^{T}x+c$$

where ***A*** is a symmetric matrix, ***b*** a vector, and ***c*** a scalar. The coefficients are estimated from a weighted least squares fit to the pixels in the neighborhood, and the weight value is related to the pixel position in the neighborhood.

The result of polynomial expansion is that each neighborhood is approximated by a polynomial. For an image frame $f_{1}$, consider the exact quadratic polynomial

(2) $$f_{1}\left(x\right)=x^{T}A_{1}x+b_{1}^{T}x+c_{1}\;$$

and construct a new image frame $f_{2}$ by a global displacement ***d***,

(3) $$\begin{array}{c} \;f_{2}\left(x\right)=f_{2}\left(x-d\right)={\left(x-d\right)}^{T}A_{1}\left(x-d\right)+b_{1}^{T}\left(x-d\right)+c_{1}\\ =x^{T}A_{1}x+{\left(b_{1}-2A_{1}d\right)}^{T}x+d^{T}A_{1}d-b_{1}^{T}d+c_{1}\;\\ =x^{T}A_{2}x+b_{2}^{T}x+c_{2}. \end{array}$$

Equating the coefficients in the quadratic polynomials yields

(4) $$\;A_{2}=A_{1},$$

(5) $$\;b_{2}=b_{1}-2A_{1}d$$

(6) $$\;c_{2}=d^{T}A_{1}d-b_{1}^{T}d+c_{1}$$

Due to the constraints:

(7) $$f_{1}\left(x\right)\approx f_{2}\left(x\right)$$

Assign

(8) $$A\left(x\right)=\frac{A_{1}\left(x\right)+A_{2}\left(x\right)}{2}$$

(9) $$\Delta b\left(x\right)=-\frac{1}{2}\left[b_{2}\left(x\right)-b_{1}\left(x\right)\right]$$

Obtain the main constraint equation:

(10) $$A\left(x\right)d\left(x\right)=\Delta b\left(x\right)\;$$

Relax the constraints and solve:

(11) $$\arg min\sum_{\Delta x\in I}w\left(\Delta x\right){\left|A\left(x+\Delta x\right)d\left(x\right)-\Delta b\left(x+\Delta x\right)\right|}^{2}$$

with the solution being:

(12) $$d\left(x\right)={\left(\sum wA^{T}A\right)}^{-1}\sum wA^{T}\Delta b$$

Assign:

(13) $$G=\sum wA^{T}A$$

(14) $$h=\sum wA^{T}\Delta b$$

In specific implementation, let w=1, then:

(15) $$G=\sum A^{T}A$$

(16) $$h=\sum A^{T}\Delta b$$

Here, ***A*** is calculated by using the characterization coefficients $r_{2}\sim r_{6}$ calculated from the two frames of images before and after, as well as the previous optical flow vector ***d***.

According to Equation (1), for the local information of an image based on binomial representation, the transformed coefficients of the image can be represented by six basis functions, defined as:

(17) $$c=r_{1},\;b=\left(\begin{array}{c} r_{2}\\ r_{3} \end{array}\right),\;A=\left(\begin{array}{cc} r_{4} & \frac{r_{6}}{2}\\ \frac{r_{6}}{2} & r_{5} \end{array}\right)$$

As a result:

(18) $$f\left(x\right)=\left(\begin{array}{cc} x & y \end{array}\right)A\left(\begin{array}{c} x\\ y \end{array}\right)+b^{T}\left(\begin{array}{c} x\\ y \end{array}\right)+c=r_{1}+r_{2}x+r_{3}y+r_{4}x^{2}+r_{5}y^{2}+r_{6}xy$$

Further expressed as: f=Br, solving the representation coefficient r is equivalent to solving the minimum linear square problem with weights

(19) $$\arg min{\left\|Br-f\right\|}_{w}$$

The solution of (19) is

(20) $$r={\left(WB\right)}^{†}Wf$$

According to the pseudo-inverse formula of ***A***,

(21) $$A^{†}={\left(A^{*}A\right)}^{-1}A^{*}$$

After expanding $\left(WB\right)$, it can be obtained that,

(22) $$r={\left(B^{*}W^{2}B\right)}^{†}B^{*}W^{2}f$$

Let $W^{2}=W_{a}W_{c}$, then

(23) $$r={\left(B^{*}W_{a}W_{c}B\right)}^{†}B^{*}W_{a}W_{c}f$$

Here, $W_{c}=I$. When implementing, set $G=B^{*}W_{a}B$

Expand to an inner product format,

(24) $$r={\left(\begin{array}{ccc} \left(a\cdot c\cdot b_{1},b_{1}\right) & \ldots & \left(a\cdot c\cdot b_{1},b_{m}\right)\\ \vdots & \ldots & \vdots \\ \left(a\cdot c\cdot b_{m},b_{1}\right) & \ldots & \left(a\cdot c\cdot b_{m},b_{m}\right) \end{array}\right)}^{-1}\left(\begin{array}{c} \left(a\cdot c\cdot b_{1},f\right)\\ \vdots \\ \left(a\cdot c\cdot b_{m},f\right) \end{array}\right)$$

After obtaining the characterization coefficient r, according to Equation (17), ***A*** can be obtained.

Then, according to (12), (15) and (16), the optical flow vector d is

(25) $$d=G^{-1}h$$

Finally, like LK sparse optical flow, we further combine the multi-layer pyramid to solve the problems of GF optical flow tracking dynamics and local minimum convergence.

### 2.3. FAST Feature Extraction

In addition to GF dense optical flow, the FPGA-based accelerator also performs FAST feature extraction. FAST is a lightweight feature point extraction algorithm. The basic idea of the algorithm is to traverse each pixel in the image and compare the pixel values of the test point with the surrounding points. If the difference between the grayscale values of this point and most other surrounding pixel points exceeds a certain range, it can be considered as a feature point. Compared to other feature point detection algorithms, FAST only needs to compare the size of pixel brightness, which is very convenient. Its calculation process is as follows [25]:

1. Select a pixel point *p* in the image and denote its brightness as $I_{p}$.
2. Set a threshold *T* for $I_{p}$.
3. With pixel point *p* as the center, select 16 pixel points on a circle with a radius of three.
4. If the brightness of *N* consecutive points on the selected circle is greater than $I_{p}+T$ or less than $I_{p}-T$ (see Equation (26)), then the pixel point *p* can be considered as a feature point.
  (26) $$\left|I_{x}-I_{p}\right|>T$$
5. Repeat the steps above to perform the same operation for each pixel.

Finally, after the above steps are completed, non-maximal suppression is generally used to preserve feature points that respond to the maximum value within a certain area to avoid the problem of excessively concentrated feature points. The non-maximum suppression method as follows: First, calculate the FAST score function value *V* of the feature points, as shown in Formula (27). If there are multiple feature points in a neighborhood centered on feature point *p*, and the score value of point *p* is the highest among all feature points in the neighborhood, then it is retained; otherwise, it is suppressed. If there is only one feature point in the neighborhood, it is retained.

(27) $$V=\left\{\begin{array}{cc} \sum \left[\left(I_{p}-I_{x}\right)-T\right] & \left(I_{p}-I_{x}\right)>T\\ \sum \left[\left(I_{x}-I_{p}\right)-T\right] & \left(I_{x}-I_{p}\right)>T \end{array}\right.$$

Together with the GF optical flow, the reserved feature points are output and written to the DDR of PS side for back-end solving.

## 3. Hardware Architecture

**Figure 2:**
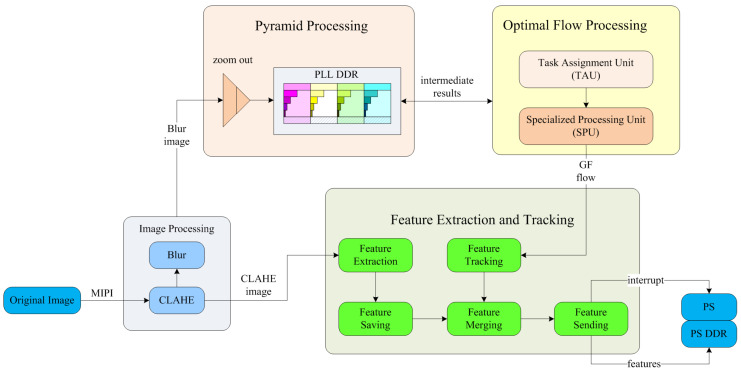
Hardware architecture of the FPGA-based accelerator for real-time visual SLAM.

Figure 2 illustrates the proposed FPGA-based feature extraction and tracking accelerator for the real-time visual SLAM system. It mainly consists of image preprocessing, pyramid processing, optical flow processing, and feature extraction and tracking modules. In this section, we introduce each of these modules one-by-one.

### 3.1. Image Preprocessing Module

Through the MIPI interface, the FPGA accelerator receives and stores the raw image stream data and performs preprocessing. First, histogram equalization (HE) is performed to extend the dynamic range of the image grayscale values to increase the image contrast. The module adopts the CLAHE algorithm with excellent performance to better solve the problem of noise amplification during image equalization.

Unlike traditional HE and AHE methods, the slope of CLAHE associated with the gray-level assignment scheme is limited, which can be accomplished by allowing only a maximum number of pixels in each of the bins associated with local histogram [26,27].

**Figure 3:**
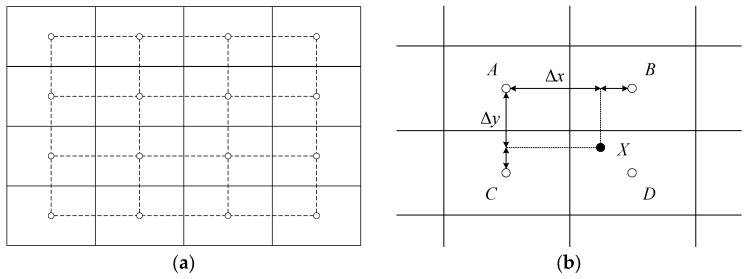
Subdivision and interpolation scheme of CLAHE. (**a**) A total of 16 contextual regions and their center points. (**b**) Bilinear interpolation using gray-levels of center points of contextual regions.

The specific implementation steps are as follows:

1. The image is divided into 16 contextual regions of size 4 × 4, and its discrete PDF can be calculated as follows:
  (28) $$\;p_{r}\left(r_{k}\right)=\frac{n_{k}}{MN},\;k=0,1,2,\cdots ,L-1$$
  where *MN* is the product of the number of rows *M* and columns *N* of image pixels, representing the total number of pixels in the image. $n_{k}$ is the number of pixels with a gray-level of $r_{k}$. *L* is the maximum number of gray-levels in the image; corresponding to an 8-bit image, the value of *L* is 256.
  On this basis, the gray-level mapping function $s_{k}$ in the contextual regions can be obtained as follows:
  (29) $$\;s_{k}=T\left(r_{k}\right)=\left(L-1\right)\sum_{j=0}^{k}p_{r}\left(r_{j}\right)=\frac{\left(L-1\right)}{MN}\sum_{j=0}^{k}n_{j},\;k=0,1,2,\cdots ,L-1$$
  where $n_{j}$ is the number of pixels with a gray-level of $r_{j}$ in the contextual region. Through the transformation of Equation (9), pixels with a gray-level of $r_{k}$ in the contextual region can be mapped to corresponding pixels with a gray-level of $s_{k}$.
2. For each sampled pixel in the image, find the points *A*, *B*, *C* and *D* from the center of the four relevant contextual regions adjacent to this pixel, with gray-level mappings $g_{A}\left(s_{k}\right)$, $g_{B}\left(s_{k}\right)$, $g_{C}\left(s_{k}\right)$, and $g_{D}\left(s_{k}\right)$, respectively, as shown in Figure 3. Assuming that the original pixel intensity at the sample point *X* is $s_{X}$, its new gray value is calculated by bilinear interpolation of the gray-level mappings that were calculated for each of the surrounding contextual regions:
  (30) $$\;s_{X}^{\prime }=\left(1-\Delta y\right)\left(\left(1-\Delta x\right)g_{A}\left(s_{k}\right)+\Delta xg_{B}\left(s_{k}\right)\right)+\Delta y\left(\left(1-\Delta x\right)g_{C}\left(s_{k}\right)+\Delta xg_{D}\left(s_{k}\right)\right)$$
  where Δx and Δy are normalized distances with respect to the pixel point *A*.

3. Set a threshold for the maximum number of pixels in each of the bins associated with local histograms, and clip and reassign pixels that exceed the threshold to limit contrast enhancement and reduce background noise. After clipping the histogram, the pixels that were clipped are equally redistributed over the whole histogram to keep the total histogram count identical. In this contribution, the clip limit is set to 3, which means that for each bin associated with the local histogram, the maximum number of pixels allowed is three times the average histogram contents.

Then, the image is filtered, which is known as Gaussian blur processing. Gaussian blur can reduce image noise, reduce the level of detail, and enhance the image effect under scales, which is conducive to the down sampling of subsequent pyramid image. A Gaussian convolution kernel with a size of 7 × 7 and a standard deviation of σ = 5 is used to perform sliding window filtering on the image. Since FPGA is not good at floating-point arithmetic, it needs to be fixed-pointed. First, the decimal portion of the Gaussian filter parameters is determined to be 12 bits. Then, the filter parameters are enlarged by 2^12^ = 4096 times by displacement and left-shifted 12 bits to retain the integer parts.

### 3.2. Pyramid Processing Module

**Figure 4:**
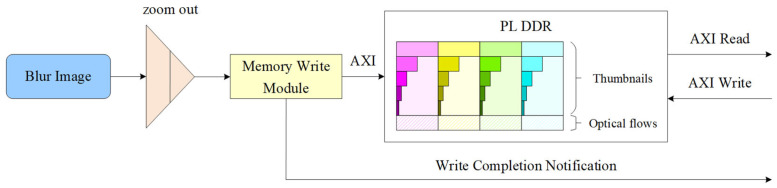
Architecture of the pyramid processing module.

The pyramid processing module receives images from the preprocessing module, zooms out the images four times with a sampling ratio of 2:1, resulting in a total of five layers of pyramid thumbnails, including the original resolution image. Afterwards, the pyramid thumbnails are synchronously output and written to the programmable logic (PL) side double date rate (DDR) synchronous dynamic random access memory (SDRAM) through the AXI interface, as shown in Figure 4.

In the PL side DDR, four consecutive frames of images (including the pyramid thumbnails) and their optical flow data are needed. This is because the FPGA receives the kth image and performs pyramid reduction, and calculates the optical flow after receiving the (*k +* 1)th image; the optical flow calculation is carried out layer by layer along the pyramid image from top to bottom, so the processing time required is

(31) $$\left(1+\left(\frac{1}{4}\right)+\left(\frac{1}{16}\right)+\left(\frac{1}{64}\right)+\left(\frac{1}{256}\right)\right)\approx 1.27\;FPS.$$

It can be seen from Equation (11) that the optical flow calculation from the (*k +* 1)th image continues until the (*k +* 2)th image. Similarly, the pyramid optical flow processing of the image received from frame (*k +* 1) will end at frame (*k +* 3). The memory and time usage during the entire pyramid optical flow processing is detailed in Table 1. It is worth noting that, unlike original images and their pyramid thumbnails, the portion of memory responsible for storing optical flow data only refreshes and stores the optical flow of the previous layer of thumbnails, thereby reducing memory size.

### 3.3. Optical Flow Processing Module

**Figure 5:**
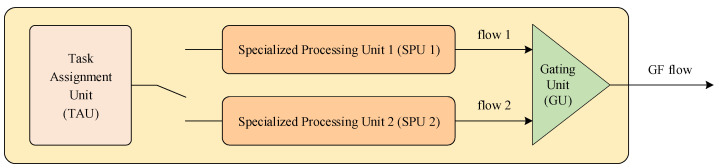
Composition of the optical flow processing module.

As can be seen from the last row in Table 1, there are at most two optical flow calculation threads simultaneously in each frame. For this reason, two specialized processing units (SPU) were designed and used in the optical flow processing module. In addition, the module includes a task assignment unit (TAU) and a gating unit (GU) for optical flow outputs, as shown in Figure 5.

The TAU is responsible for obtaining the status of the two SPUs and finding out which one is idle. Once the external notification signal is received (see Figure 5), the TAU sends a start signal with the group address serial number to the idle SPU.

**Figure 6:**
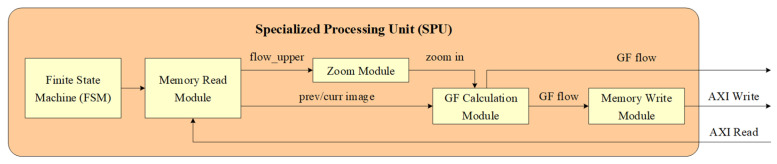
Detailed structure of the SPU.

The detailed structure of the SPU is shown in Figure 6. The finite state machine (FSM) calculates all addresses based on the group address serial number, and then sequentially starts state processing for the 5-layer pyramid thumbnail. The state processing of each layer includes sending memory read commands and waiting for the optical flow calculation to complete. After the state processing of a layer is completed, the FSM switches to the next layer state.

The memory read module simultaneously reads three channels of data from the PL side DDR: the previous frame image, the current frame image, and the previous layer of optical flow data. The zoom module receives the previous layer of optical flow data and zooms in two times to output. The zoom in operation adopts a bilinear interpolation method, which executes the horizontal direction first, and then the vertical direction. The GF calculation module receives the previous and current frame images, as well as optical data from the zoom module, for GF dense optical flow calculation. The GF optical flow is written into the PL side DDR through the memory write module for the optical flow calculation of the next frame image and is also directly output to the feature extraction and tracking module.

For the two optical flow output pipelines SPU 1 and SPU 2, only one will be valid at the same time. Therefore, the GU distinguishes the output line through the valid signal and outputs the GF optical flow calculation result.

**Figure 7:**
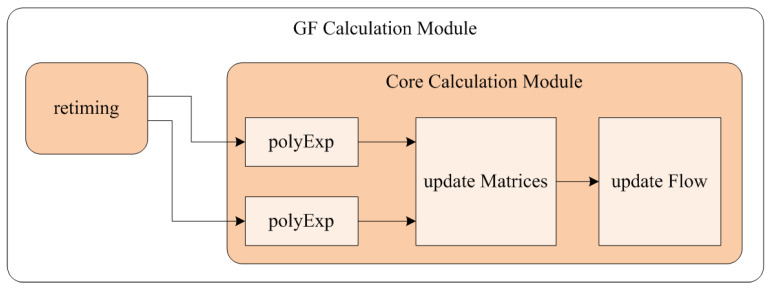
Architecture of the GF calculation module.

The architecture of the GF calculation module is shown in Figure 7. The retiming module sends read commands to the memory module, generating HSYNC and VSYNC. The polyExp module calculates the coefficients $r_{1}\sim r_{6}$ in Equation (18). According to Equations (15) and (16), the update Matrices module calculates the components of G and h. The update Flow module smoothes the various components of G and h, and then calculates the optical flow vector ***d*** according to Equation (25).

### 3.4. Feature Extraction and Tracking Module

The feature extraction and tracking module is responsible for extracting and recording FAST features, and calculating their coordinates in the new image according to the optical flow results. Moreover, it writes the reorganized feature point array into the PS side DDR and sends an interrupt signal to the PS side after completion.

**Figure 8:**
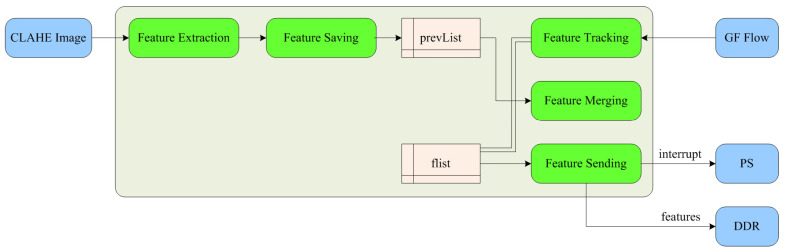
Architecture of the feature extraction and tracking module.

**Figure 9:**
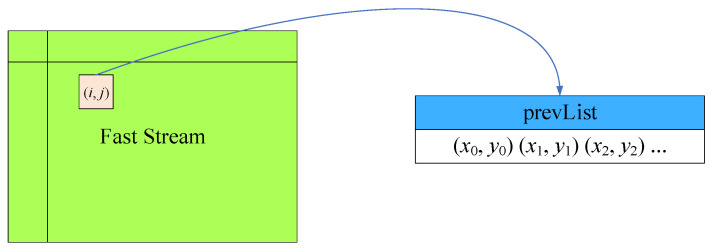
Coordinate storage of features.

The composition of the feature extraction and tracking module is shown in Figure 8, including that of feature extraction, feature saving, feature tracking, feature merging, and feature sending submodules. First, the module receives the CLAHE-processed image and performs FAST feature extraction. Secondly, the feature saving submodule receives the FAST feature data of stream type through the AXI interface, and reads each item of the stream data by line buffer. If a value other than 0 is read in the line buffer, it indicates that the corresponding point in the image is a valid feature, and the image coordinates of the feature are saved in the array pointer “prevList”, as shown in Figure 9. Due to the need for ping-pong read–write operations, “prevList” needs to be neutralized in BRAM by two copies, defined as “prevList 1” and “prevList 2”, respectively.

**Figure 10:**
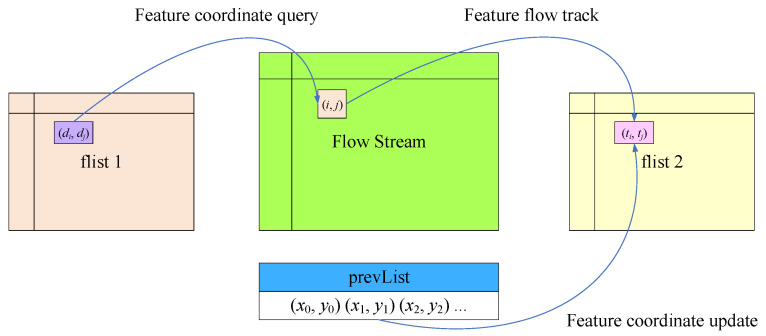
Optical flow tracking and coordinate updating of features.

The feature tracking submodule receives the stream type GF optical flow data through the AXI interface and converts it into mat format. It then defines an array pointer “flist” to store the abbreviated coordinates of FAST features updated by optical flow tracking. Similarly, due to the need for ping-pong read–write operations, “flist” needs to be neutralized by 2 copies, defined as “flist 1” and “flist 2”, respectively. As shown in Figure 10, the left array pointer “flist 1” stores the coordinates corresponding to features of the previous frame of the image; in the middle is the full-frame optical flow data currently being output pixel by pixel, which is read by line buffer. Since the position of features in the previous frame of the image is known, the specific positions of features in the optical flow can be determined in advance.

When the full-frame optical flow data is output to these positions, the feature merging submodules reads the feature coordinates from “prevList”, uses the optical flow to track features, obtains the position of features in the next image, and stores the updated feature coordinates in the array pointer “flist 2” on the right. Finally, the feature sending submodule sends the feature ID and coordinate data from “flist 2” to the DDR, and generates an interrupt signal after completion.

## 4. Evaluation and Discussion

The implementation of the proposed accelerator on a Xilinx Zynq FPGA is described in this section. Further, in order to verify the feasibility and performance of the proposed solution, a test benchmark system was built and evaluation work was carried out.

### 4.1. Test Benchmark System

The proposed feature extraction and tracking accelerator was implemented on a modern Xilinx Zynq FPGA (UltraScale+ MPSoC ZU15EG), a device that combines abundant hardware resources on a single chip. Hardware programmability allows a direct, lowest-level interface to the CMOS sensor, enabling a convenient and reliable image acquisition process. Moreover, to verify the feasibility and performance of the accelerator, a test benchmark system was built. It consists of the FPGA accelerator, as well as a PC. Compared with the official version, the FPGA accelerator in the test benchmark system was slightly modified in terms of command reception and memory storage to enable comparison and verification with the PC.

**Figure 11:**
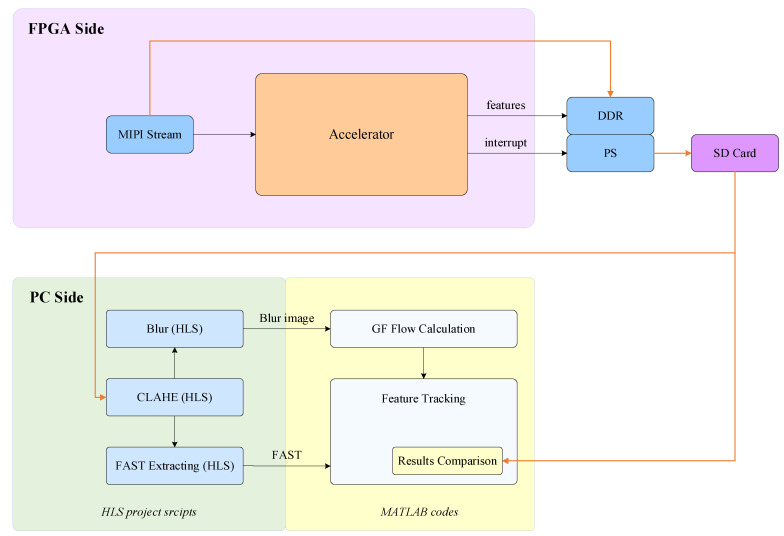
Workflow of the test benchmark system.

The overall workflow of the test benchmark system is shown in Figure 11. It mainly includes the following operations:

1. FPGA side: receive the MIPI image, and perform the GF optical flow calculation and the FAST feature extraction and tracking processing.
2. FPGA side: store the FAST feature tracking result and original image data in the PS side DDR, and transfer them to an SD card.
3. PC side: read the raw image data from the SD card, and obtain intermediate results on image preprocessing and FAST feature extraction through high-level synthesis (HLS) simulation [24,28].
4. PC side: input the intermediate results to the MATLAB-based optical flow calculation and feature tracking program to obtain the FAST feature tracking results of MATLAB.
5. Compare feature tracking results from FPGA and MATLAB for verification and analysis.

### 4.2. Evaluation Results

**Figure 12:**
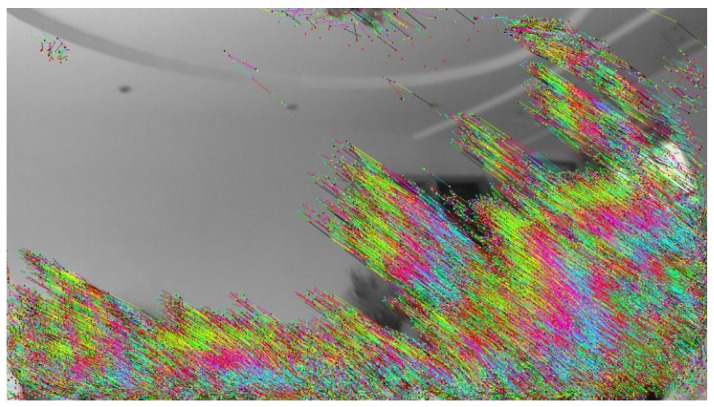
FAST feature tracking results based on dense optical flow. The processing results of the FPGA accelerator and the MATLAB benchmark completely coincide.

The proposed accelerator was implemented on a Xilinx Zynq FPGA (UltraScale+ MPSoC ZU15EG). The camera was shaken vigorously to continuously acquire images, and the test benchmark system was used to analyze and evaluate the processing results of the accelerator. A continuous 64 frames of images were tested; the operation results show that the accelerator could stably track the features of severely shaking images, and the processing results were 100% consistent with those from MATLAB on the PC end. Figure 12 presents the dense optical flow calculation and FAST feature tracking results for one of these frames. In the figure, we use different colors to represent the motion of each frame, and the image shows the superposition of motion trajectories of 64 frames. In can be seen that the processing results from the FPGA accelerator are the same as the processing results from the MATLAB benchmark, which proves the effectiveness and correctness of the proposed real-time V-SLAM accelerator.

### 4.3. FPGA Resource Consumption

The proposed accelerator consumes 123,300 LUTs (look up table), 386.5 M bytes of BRAMs, 68 M bytes of URAMs, and 686 DSPs. The specific hardware consumptions and occupations are detailed in Table 2. For 720P resolution images (1280 × 720), it can process 100/(1280 × 720) = 108 frames of images per second; for 1080P resolution images (1920 × 1080), it can process 100/(1920 × 10,800) = 48 frames of images per second. The accelerator can operate up to more than 200 MHz, further doubling the processing power.

We compared the proposed hardware with the PC-based AMD multi-core CPU. Both are based on a 1280 × 720 resolution image for dense optical flow calculation and FAST feature extraction and tracking. For the proposed hardware (operating at 100 MHz), each frame takes (1280 × 720)/100 MHz ≈ 10 ms; for MATLAB code running on the Ryzen R7 6800H clocked at 3.5 GHz, each frame takes about 17 s.

### 4.4. Complexity Analysis

The module with the highest computational complexity in the accelerator scheme is the GF dense optical flow calculation module. The specific computational complexity will be analyzed below.

① According to Equations (17) and (18), A and b are calculated through six sets of filters.
  For each point, perform six sets of 11 × 11 2D filtering, with 22 multiply−accumulate(MAC), 7 multiplications, and 2 additions, resulting in a computational cost of
  (32) 22MAC+7×Mult+2×Add
  where MAC is equivalent to 11 multiplications and 19 additions. After calculating both frames simultaneously,
  (33) $${\;N}_{p}\times 2=6\times 44\times \left(11\times Mult+19\times Add\right)+14\times Mult+4\times Add\;\;=2918\times Mult+5020\times Add$$
② Calculate G and h using the front and back frames as well as the upper optical flow:
  (34) $$N_{Gh}=12\times Mult+14\times Add$$
③ Perform 15 × 15 box filtering on G and h. Each pixel position corresponds to five sets of 15× 15 box filtering of 32 MACs, 8 multiplications, 3 additions, and 1 division, and the calculation amount for each pixel is:
  (35) $$N_{f}=5\times 32MAC+8\times Mult+3\times Add+1\times Div$$
  where multiply accumulate(MAC) is equivalent to 29 additions; then,
  (36) $$N_{f}=5\times 30\times 29Add+8\times Mult+3\times Add+1\times Div\;\;=8\times Mult+4353\times Add+1\times Div$$
  Therefore, the total amount of computation for each pixel is:
  (37) $$N_{s}=N_{p}\times 2+N_{Gh}+N_{f}\;\;=2938\times Mult+9387\times Add+1\times Div$$
  The number of pixels processed by the accelerator per second is:
  (38) $$N_{p}=1280\times 720\times 60=55,296,000$$
  Therefore, the total computation per second of the accelerator is:
  (39) $${{N_{c}=N}_{s}\times N}_{p}=0.68TOPS$$
  where TOPS means tera operations per second.

### 4.5. Limitations

The FPGA-based accelerator scheme proposed still has certain limitations. On the one hand, in FPGA implementation, fixed-point computation was used due to the high utilization of floating-point computing resources, which may lead to computational accuracy issues and overflow issues. In addition, due to the size limitation of the Bram, only 8 rows above and below can be updated in the optical flow update section, which to some extent reduces the accuracy and stability of optical flow tracking.

## 5. Conclusions

An FPGA-based feature extraction and tracking accelerator for real-time VIO and Visual SLAM application is presented, which could realize the complete acceleration processing function of the image front-end and directly output the feature point ID and coordinates to the back-end. The accelerator performs CLAHE and Gaussian blur for image preprocessing. For the first time, it implements a solution that combines FAST features with GF dense optical flow to achieve better feature tracking performance and provide more flexible technical route selection for the back-end. In order to solve the scale invariance and rotation invariance lacking problems of FAST features, a pyramid module with a five-layer thumbnail structure was designed and implemented, and the pipeline and memory read and write operations were optimized.

The proposed accelerator was implemented on a Xilinx Zynq FPGA (UltraScale+ MPSoC ZU15EG). The evaluation results based on the test benchmark system show that the accelerator could achieve stable tracking of features of violently shaking images, and were consistent with the processing results of the MATLAB code on the PC side. It consumes 36% of the LUTs, 52% of the BRAM, and 19% of the DSP of the Zynq FPGA. When operating at 100 MHz, the accelerator can process 108 frames per second for 720P images and 48 frames per second for 1080P images. Compared to PC CPUs, which require seconds of time for processing, the processing latency is greatly reduced to the order of milliseconds, making GF dense optical flow an efficient and practical technical solution on the edge side.

## Figures and Tables

**Table 1:** Memory and time usage of the pyramid processing module.

	Sequences				
	*i* − 1	*i*	*i* + 1	*i* + 2	*i* + 3
Memory content	The (*k* − 1)th image and its pyramid thumbnails;	The (*k* − 1)th image and its pyramid thumbnails;	The (*k* − 1)th image and its pyramid thumbnails;	The optical flow between the (*k* − 2)th and (*k* − 1)th images;	
		The *k*th image and its pyramid thumbnails;	The *k*th image and its pyramid thumbnails;	The *k*th image and its pyramid thumbnails;	The optical flow between the (*k* − 1)th and *k*th images;
			The (*k +* 1)th image and its pyramid thumbnails;	The (*k +* 1)th image and its pyramid thumbnails;	The (*k +* 1)th image and its pyramid thumbnails;
				The (*k +* 2)th image and its pyramid thumbnails;	The *(k +* 2)th image and its pyramid thumbnails;
					The (*k +* 3)th image and its pyramid thumbnails.
Processing tasks	Receive the (*k* − 1)th image and perform pyramid down;	Calculate the pyramid optical flow of the (*k* − 1)th image;	Obtain the pyramid optical flow of the (*k* − 1)th image;	Obtain the pyramid optical flow of the *k*th image;	Obtain the pyramid optical flow of the (*k +* 1)th image;
		Receive the *k*th image and perform pyramid down;	Calculate the pyramid optical flow of the *k*th image;	Calculate the pyramid optical flow of the (*k +* 1)th image;	Calculate the pyramid optical flow of the (*k +* 2)th image;
			Receive the (*k +* 1)th image and perform pyramid down;	Receive the (*k +* 2)th image and perform pyramid down;	Receive the (*k +* 3)th image and perform pyramid down
				Calculate the optical flow between the (*k* − 1)th and *k*th images;	Calculate the; optical flow between the *k*th and (*k +* 1)th images.

**Table 2:** Hardware consumption of the proposed FPGA accelerator.

Resource	Available	Utilization	Utilization %
LUT (look up table)	341,280	123,300	36%
FF (flip flop)	682,560	147,172	22%
BRAM	744	386.5	52%
URAM	112	68	61%
DSP	3528	686	19%
IO	328	82	25%
BUFG	404	15	4%
MMCM	4	1	25%
PLL	8	3	38%

## Data Availability

The data that support the findings of this study are available from the corresponding author upon reasonable request.
